# Contextual exploration previous to an aversive event predicts long-term emotional consequences of severe stress

**DOI:** 10.3389/fnbeh.2013.00134

**Published:** 2013-10-02

**Authors:** Carlos E. N. Girardi, Paula A. Tiba, Gisela B. Llobet, Raquel Levin, Vanessa C. Abilio, Deborah Suchecki

**Affiliations:** ^1^Departamento de Psicobiologia, Universidade Federal de São PauloSão Paulo, Brazil; ^2^Centro de Matemática, Computação e Cognição, Universidade Federal do ABCSanto André, Brazil; ^3^Departamento de Farmacologia, Universidade Federal de São Paulo São Paulo, Brazil; ^4^Departamento de Psiquiatria, Laboratório Interdisciplinar de Neurociências Clínicas, Universidade Federal de São PauloSão Paulo, Brazil

**Keywords:** context fear conditioning, contextual configuration, PTSD, emotional behavior, psychiatric disorder, animal model

## Abstract

Traumatic stress can lead to long-term emotional alterations, which may result in Posttraumatic Stress Disorder (PTSD). Fear reactions triggered by conditioned cues and exacerbated emotional arousal in face of non-conditioned stimuli are among the most prominent features of PTSD. We hypothesized that long-term emotional alterations seen in PTSD may depend on the strength of context-trauma association. Here, we investigated the contribution of previous contextual exploration to the long-term emotional outcomes of an intense foot shock in rats. We exposed male Wistar rats to a highly stressful event (foot shock, 2 mA, 1 sec) allowing them to explore or not the chamber prior to trauma. We, then, evaluated the long-term effects on emotionality. Fear was assessed by the time spent in freezing behavior either upon re-exposure to trauma context or upon exposure to an unknown environment made potentially more aversive by presentation of an acoustic stimulus. Behaviors on the elevated-plus-maze and acoustic startle response were also assessed. The possibility to explore the environment immediately before the aversive event led to differential long-term emotional effects, including a heightened freezing response to re-exposure to context, blunted exploratory behavior, fear sensitization and exacerbation of the acoustic startle response, in contrast to the minor outcomes of the foot shock with no prior context exploration. The data showed the strong contribution of contextual learning to long-term behavioral effects of traumatic stress. We argue that contextual representation contributes to the robust long-term behavioral alterations seen in this model of traumatic stress.

## Introduction

Traumatic situations involve intense acute stress that can exceed the adaptive capacity of an individual. The lifetime prevalence of exposure to traumatic events can be as high as one third of the population (Breslau et al., 1991), and some are likely to develop Posttraumatic Stress Disorder (PTSD), an anxiety disorder characterized by flashbacks, hyperarousal, reactions of avoidance and numbing that start approximately 6 months after the trauma and persist for at least a month or more (DSM-IV, 2000).

The prevalence of PTSD among victims of trauma is 20 to 30% (Breslau et al., 1991, 1998). The fact that not all victims of traumatic events develop PTSD prompts the interest for elucidation of factors that may be involved in individual resilience or vulnerability. Knowledge about susceptibility factors in psychiatric disorders is limited to retrospective studies based on subjective information on the life history of patients. Consequently, controlled studies with planned manipulations of factors likely related to emotional alterations stemming from traumatic stress can exclusively be performed in laboratory animals. PTSD basic research is focused mainly in rodent models in which long-term associative and non-associative behavioral alterations (as conditioned fear, hyperarousal, blunted emotionality and exaggerated startle response) are assessed following a strongly aversive experience that is considered to be traumatic (Charney et al., 1993; Yehuda and Antelman, 1993; Gewirtz et al., 1998; Sanford et al., 2003; Jha et al., 2005; Pawlyk et al., 2005; Siegmund and Wotjak, 2006, 2007a,b; Golub et al., 2009; Clay et al., 2011; Golub et al., 2011; Pamplona et al., 2011).

Among the myriad of outcomes of a traumatic event, recurrent recall of the traumatic experience is strongly related to the occurrence of PTSD symptoms, through flashbacks, exaggerated response to trauma cues and intrusive nightmares (DSM-IV, 2000). Re-experiencing of trauma-related memories may underlie the arousing symptoms of PTSD (Cannistraro and Rauch, 2003). In fact, it has been under debate whether PTSD is actually a memory disorder, in which a failure to process the traumatic information and properly integrate it into preexisting memory systems would prompt the maladaptive and persistent distress reactions that compose the debilitating symptoms seen in PTSD patients (Witvliet, 1997; Elzinga and Bremner, 2002). It has been suggested that, deficits in contextual processing of the traumatic memories may underlie the inappropriate behavioral responses that characterize the development of PTSD (Rougemont-Bucking et al., 2011; Maren et al., 2013).

Although it is not possible to assess spontaneous memory recall in non-human animals, reactions triggered by implicit memory retrieval are experimentally induced in animal studies using the fear conditioning paradigm (Sanford et al., 2003; Jha et al., 2005; Pawlyk et al., 2005; Siegmund and Wotjak, 2006, 2007a,b; Siegmund et al., 2009; Golub et al., 2011; Pamplona et al., 2011). Fear conditioning is the associative learning between an aversive stimulus (typically an electric foot shock in rodents) and discrete cues or an assembly of elements that are emotionally neutral, named conditioned stimuli (Rescorla, 1968; Fanselow, 1984). When placed in the novel environment, the experimental animal initiates an active process of exploratory behavior. Delivering a foot shock after environmental exploration leads to an associative process (fear conditioning), in which fear is further expressed upon another exposure to the previously paired cues, with no shock presentation.

For contextual fear conditioning, the assembly of multimodal cues present in the environment is integrated during the exploratory process, characterizing the context into a unified contextual representation (Sanders et al., 2003; Rudy et al., 2004). In animal studies of PTSD, re-exposure to the paired environment is frequently employed to trigger detectable emotional responses that are believed to resemble, at least in basic aspects, the symptoms manifested in PTSD patients (Jha et al., 2005; Pawlyk et al., 2005; Siegmund and Wotjak, 2007a,b; Golub et al., 2009; Pamplona et al., 2011). It has been well-established in the fear conditioning literature, that more than 20 sec of pre-shock context exploration are necessary to elicit a robust behavioral conditioned fear response during re-exposure (Fanselow, 1986; Landeira-Fernandez et al., 2006). When the shock is delivered immediately after placement of the animal in the context, with insufficient environmental exploration, conditioning is not effective, and this phenomenon is coined as immediate shock deficit. Therefore, environment familiarization prior to foot shock contributes to the strength of the fear response expressed upon re-exposure to the conditioned context (Fanselow, 1986, 1990; Frankland et al., 2004).

One question that arises from the above assumptions is: given that the duration of prior environmental exploration determines the amount of conditioned fear expressed afterwards, would the strength of the contextual memory also influence the non-associative deleterious outcomes of the aversive experience? To address this question, we applied the immediate shock paradigm, in order to test the contribution of context familiarization before foot shock to long-term fear sensitization, exploratory behavior and exaggerated acoustic startle response in a fear conditioning-based animal model of traumatic stress.

## Materials and methods

### Subjects

Thirty male Wistar rats, 90–120 days of age, were obtained from the Center for Development of Experimental Models (CEDEME), Universidade Federal de São Paulo. Rats were kept under controlled light (12 h light-dark cycle, lights on at 7:00 a.m.) and temperature (23 ± 2°C) and housed in groups of five per cage. Tests were performed between 2:00 and 5:00 p.m., i.e., during the light phase of the cycle. All procedures were approved by, and conducted in accordance with the Research Ethics Committee of Universidade Federal de São Paulo, approval protocol #2083/08.

### Stress paradigm

The aversive event, considered as the traumatic stress, was an intense and inescapable electric foot shock (2 mA, 1 sec) carried out inside a fear-conditioning chamber. The chamber was made of black walls with transparent acrylic cover which base consisted of stainless steel rods (0.4 cm diameter, spaced 1.2 cm apart), wired to a source of electric shock and scrambler (AVS Instruments, São Paulo, Brazil). Rats were randomly assigned to three groups, containing 10 subjects each, named: delayed shock (DS), immediate shock (IS) and no shock (NS). Rats in the DS group could explore the environment for 2 min before shock delivery, which had the potential to increase the likelihood that they would gain contextual information of the conditioning chamber. After this period, the electric current generator was activated. For the IS group, foot shock was released instantly upon placement of the rat into the chamber; thus, there was no opportunity to explore the environment, reducing the likelihood of gaining enough knowledge about characteristics of the conditioning chamber (Siegmund and Wotjak, 2007b; Siegmund et al., 2009; Golub et al., 2011). The rats from both groups were removed from the chamber immediately after shock delivery. NS rats were given the opportunity to explore the conditioning chamber for 2 min with no shock delivery. After the procedure, each rat was kept in an individual cage until all rats from the same cage underwent the procedure. After that they were all brought back to home cage.

### Re-exposure

Re-exposure to the conditioning chamber occurred 14 days after the conditioning, for 5 min, with no shock delivery. To evaluate the expression of fear upon re-exposure to the context, we scored freezing behavior, a defensive behavior defined as complete absence of movement, except for breathing-related motion (Blanchard et al., 1986). Scoring was performed online by an experimenter blind to the experimental conditions. Outcome data was expressed as percentage of time spent in freezing behavior, in seconds, as follows: %time freezing = (freezing time/300) × 100. In order to avoid conditioned response to the pre-stress elements, we used a distinct transportation cage (different material, color and size) and the animals were transported to the experimental room by an unfamiliar experimenter.

### Elevated plus maze

Exploratory behavior was assessed on the elevated plus maze (EPM), which is elevated 60 cm from the floor and consists of four arms: two arms enclosed by 30 cm high walls and two open arms (with no enclosing walls). Each arm is 50 cm long by 10 cm wide. Procedures were based on those described elsewhere (Pellow et al., 1985). All rats were brought to the experimental room 12 h before the test, housed in their own home cage. The test was carried out under dimmed light conditions (10 lux) and each rat was placed individually in the central compartment of the maze facing an open arm (either left or right, randomly selected) and left alone to explore the maze for 5 min. The whole procedure was video recorded using a digital camera for offline analysis. We assessed the number of entries and percentage of time spent in each arm of the EPM using the X-Plo-Rat software, developed by Khallil Taverna Chaim and Silvio Morato from the Laboratory of Exploratory Behavior, Department of Psychology and Education FFCLRP, Universidade de São Paulo, Ribeirão Preto, São Paulo, Brazil.

Arm entries were considered when the rat crossed the line between the arm and the central square with all four paws. The whole apparatus was virtually divided by equally sized squares (10 cm × 15 cm). Data are presented as the number of entries in each arm (closed or open) as well as the number of squares traveled in each arm. In order to complement exploratory activity data, we also evaluated immobility on the EPM. We chose to score immobility because the analysis was performed offline on recorded videos which quality was not sufficient to assess freezing precisely. Immobility was defined as any moment in which rats were not moving on the maze, but not necessarily in freezing. Data are presented as total immobility time during the 5 min test.

### Non-associative fear

Fear expressed upon presentation of an unknown and potentially aversive stimulus was evaluated in a 60 cm diameter cylindrical arena, divided into three concentric circles, each subdivided in segments. Each rat was placed individually in the center circle of the arena and allowed to freely explore the environment for 5 min. Besides representing an unknown situation which can be aversive by itself, the arena has been made potentially more aversive by the presence of four 60-watt bulbs lit for the entire test and the presentation of a discrete pulse noise of 90 dB with a duration of 10 sec on two occasions during the last 2 min of the test, apart by a 50 sec interval. The time of freezing behavior was assessed, both before (first 3 min) and during the presentation of sound stimuli (last 2 min). Freezing assessment was done online by an experimenter blind to the experimental conditions. The whole procedure was video recorded using a digital camera for offline analysis of ambulation during the 3 min period before sound presentation. This data is presented as number of total segments ambulated and number of traveled segments in central and peripheral areas.

### Acoustic startle response

To assess startle response to an acoustic pulse we used a sound-proof startle chamber (Insight^®^ Scientific Equipment), in which the amplitude of startle was measured after the presentation of acoustic stimuli (pulses). During the entire test there was a 65 dB background noise. Rats were acclimatized inside the startle chamber for 5 min on the day before the test and for the first 5 min during the test. Acoustic stimuli were randomly presented 30 times, separated by random intervals of ± 20 sec. Measurements were taken randomly during the 30 pulses of 120 dB of 50 ms (capable of inducing a startle response). Additionally, another 10 measurements were taken, in absence of stimuli. Results are presented as the difference between mean startle amplitude averaged among all trials when the stimulus was presented and mean startle amplitude (MSA) averaged among trials when the stimulus was absent, as follows: Δ Startle amplitude = MSApulse present − MSApulse absent.

### Experimental design

**Figure 1:**
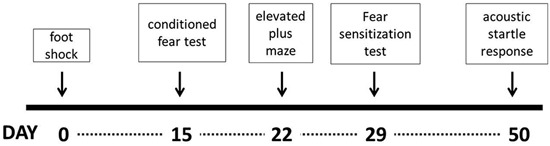
**Graphical representation of the time course of the experimental design**.

The experiment started with the electric foot shock procedure, which represented the traumatic event. Assessment of contextual fear was performed 14 days later and the remaining tests (elevated plus maze, fear to discrete stimulus and acoustic startle response, respectively) were carried out sequentially at intervals of 7 days, except for the acoustic startle response, which was performed 21 days after the previous test (Figure 1). All parameters were scored and analyzed only by one experimenter blind to all conditions (Carlos Eduardo Neves Girardi).

### Statistical analysis

Most parameters (freezing at re-exposure, elevated plus maze data and Δ MSA) were analyzed by one-way ANOVA for comparison among the experimental groups (NS, IS and DS). Comparison of freezing behavior during the re-exposure to the context (min 1, min 2, min 3, min 4 and min 5) as well as before and during sound presentation in the open arena was done by repeated measures ANOVA. Analysis of pairwise differences was carried out by Newman-Keuls *post hoc* test. The significance level for statistical difference was set at *p* ≤ 0.05.

## Results

### Re-exposure

**Figure 2:**
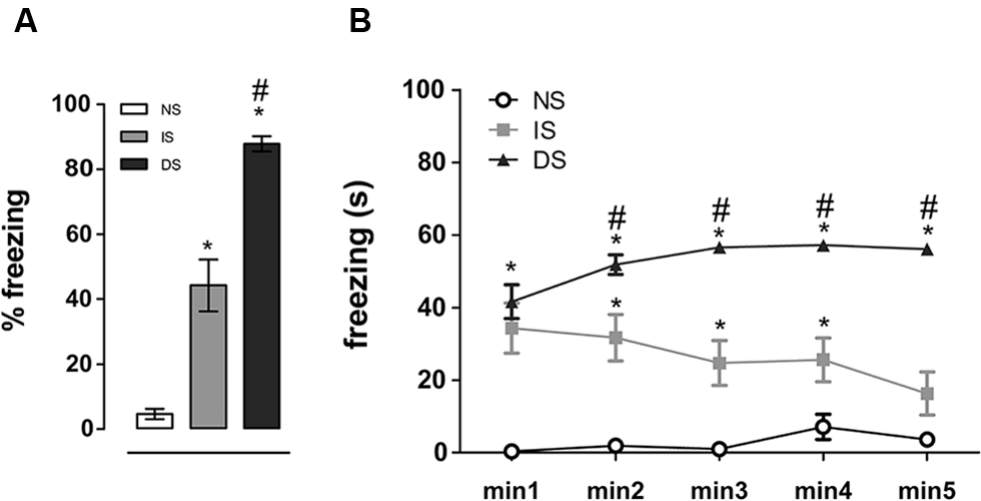
**Freezing behavior upon re-exposure to the trauma context**. Total percentage **(A)** and freezing time expressed minute by minute of exposure time **(B)**. NS = no-shock group (*n* = 10); IS = immediate shock group (*n* = 10); DS = delayed shock group (*n* = 10). *p < 0.05 compared to NS group; ^#^*p* < 0.05 compared to IS group.

Re-exposure to the conditioning chamber elicited freezing behavior in shocked animals, indicated by a main effect of group (*F*(2, 27) = 72.906, *p* < 0.001). *Post-hoc* analyses revealed that both DS (*p* < 0.001) and IS (*p* < 0.001) groups had higher percentage of freezing compared to NS group and that DS group exhibited substantially higher freezing than the IS group (*p* < 0.001) (Figure 2A). When a minute by minute analysis was performed, repeated measures ANOVA detected a significant effect for group (*F*(2, 27) = 72.897, *p* < 0.001), no effect for minute (*F*(4, 27) = 1.183, *p* = 0.322) and a significant interaction group × min (*F*(8, 108) = 4.303, *p* = 0.001). *Post-hoc* comparisons revealed that DS group had higher freezing then NS group throughout the whole session (*p* < 0.001 at all time-points), and higher freezing than IS group from the second minute on (*p* = 0.209 at minute 1, 0.004 at minute 2 and < 0.001 at the other minutes). The IS group expressed more freezing on the beginning of the re-exposure session which lowered throughout the session, reaching NS levels in the end (Figure 2B).

### Elevated plus maze

#### Arm exploration

**Figure 3:**
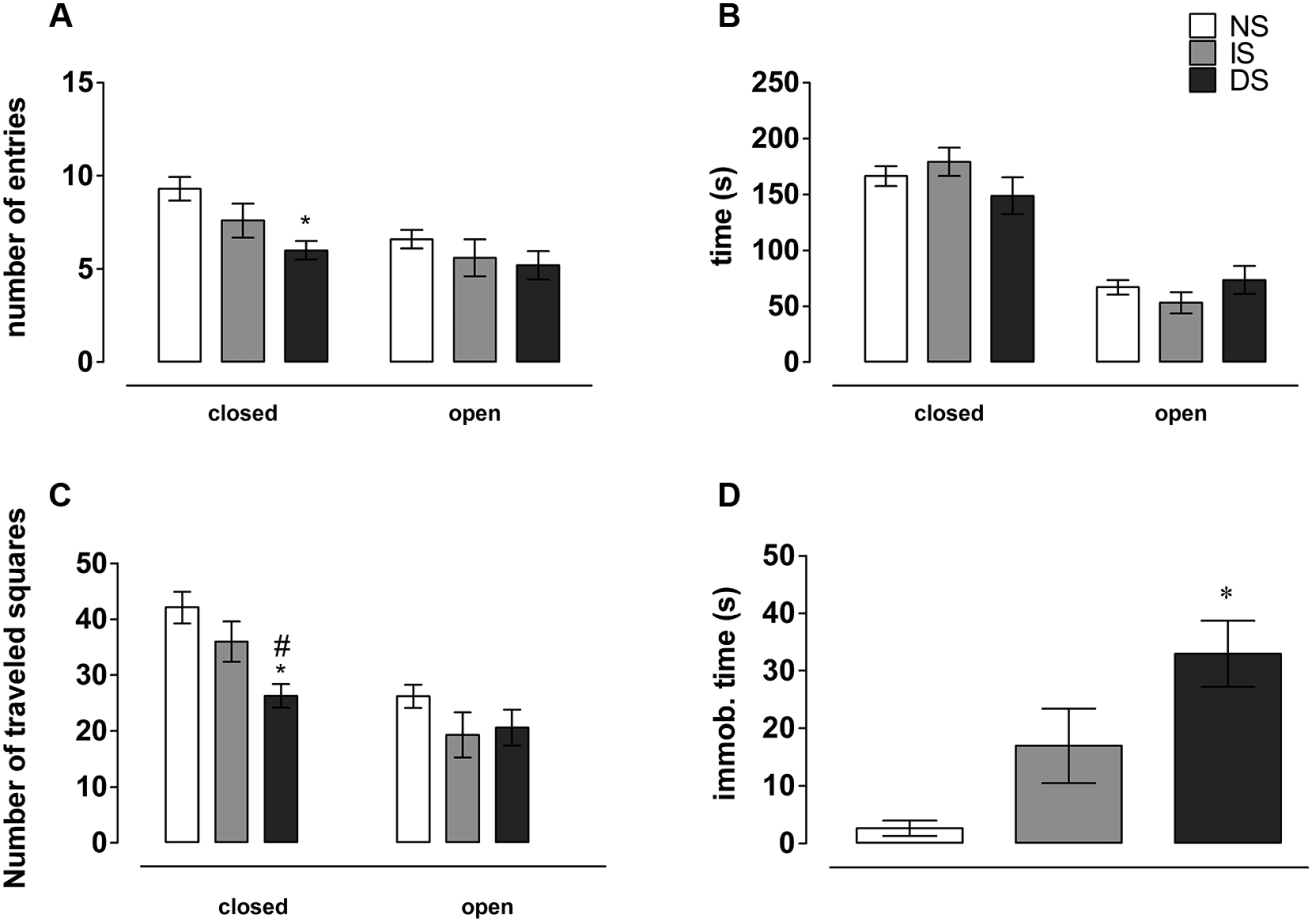
**Behavior on the Elevated Plus Maze (EPM).** **(A)** Number of entries in the different EPM compartments; **(B)** time spent exploring maze arms. **(C)** Number of traveled squares by compartment; **(D)** immobilization time on the EPM. NS = no-shock group (*n* = 10); IS = immediate shock group (*n* = 10); DS = delayed shock group (*n* = 10). *p < 0.05 compared to NS group; ^#^*p* < 0.05 compared to IS group.

There was no significant effect on open (*F*(2, 27) = 0.864, *p* = 0.430), but a significant effect on closed arms entries on the EPM (*F*(2, 27) = 5.466, *p* = 0.01). *Post-hoc* analysis showed that DS rats entered less in the closed arms than NS rats (*p* = 0.007), with IS group being undistinguishable from both DS (*p* = 0.120) and NS groups (*p* = 0.100) (Figure 3A). There was no significant difference for the time spent in the open (*F*(2, 27) = 1.133, *p* = 0.337) or in the closed arms of the EPM (*F*(2, 27) = 1.383, *p* = 0.268) (Figure 3B).

#### Traveled squares

There was a significant effect on the number of squares traveled in the closed (*F*(2, 27) = 7.456, *p* = 0.002), but not in the open arms (*F*(2, 27) = 1.299, *p* = 0.289) of the EPM. *Post-hoc* analysis showed that DS rats crossed fewer squares in the closed arms than NS (*p* = 0.002) and IS rats (*p* = 0.026), without any difference between IS and NS rats (*p* = 0.151) (Figure 3C).

#### Immobility

For immobility time on the elevated plus maze, there was a significant difference (*F*(2, 27) = 8.976, *p* = 0.001). *Post-hoc* analysis showed that DS rats spent more time in immobility than NS group (*p* < 0.001). DS also spent more time in immobility than IS group (*p* = 0.034). The difference between IS and NS groups was nearly significant (*p* = 0.055) (Figure 3D).

### Non-associative fear

**Figure 4:**
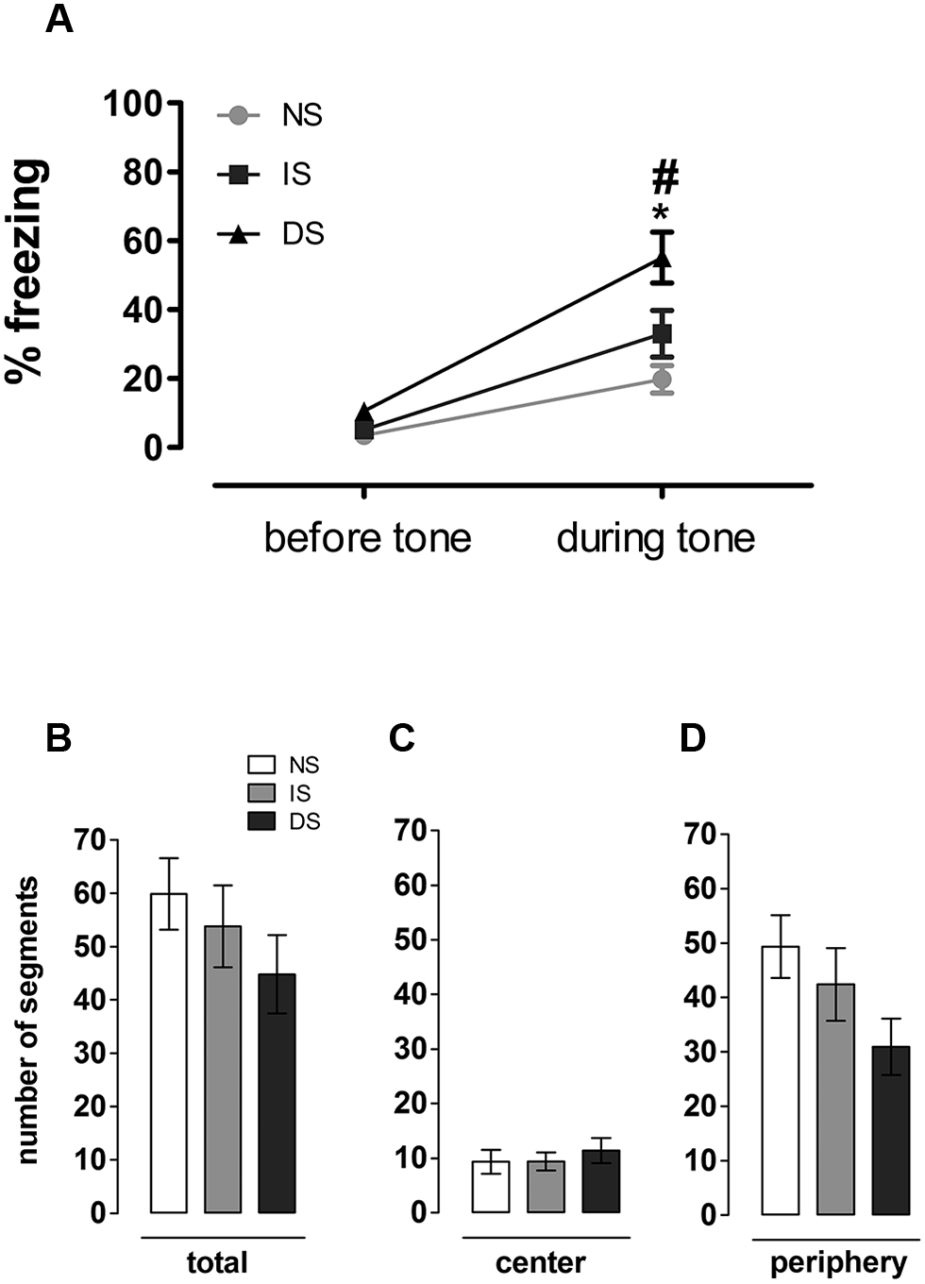
**Open field analyzes**. **(A)** Expression of fear in a mildly aversive situation. Percentage of time in freezing before and during tone presentation. Number of **(B)** total **(C)** central and **(D)** peripheral segments traveled. NS = no-shock group (*n* = 9); IS = immediate shock group (*n* = 10); DS = delayed shock group (*n* = 10).

For this test, one animal was not included in the analysis due to technical problems with video recording. There was a significant effect of group (*F*(2, 26) = 7.612, *p* = 0.002), of sound (*F*(1, 26) = 76.699, *p* < 0.001) and a group × sound interaction (*F*(2, 26) = 5.051, *p* = 0.014) on the percentage of freezing behavior in the open field. *Post-hoc* analyses revealed no differences among groups before sound presentation (*p* = 0.850 (NS × IS); 0.590 (NS × DS); 0.430 (IS × DS)); yet the two subsequent minutes following sound presentation were marked by a considerable group effect in fear expression: while NS and IS animals reacted to sound by a discrete increase in freezing behavior (evidenced by the overall sound effect), DS group showed considerably higher percentage of freezing compared to itself before (*p*) and to NS (*p* < 0.001) and IS (*p* = 0.05) during sound presentation. Upon sound presentation, NS and IS did not differ from each other (*p* = 0.677) (Figure 4A). There was no significant difference in the number of traveled segments in the open field (*F*(2,26) = 1.067; *p* = 0.358) (Figure 4B), nor when central (*F*(2,26) = 0.329; *p* = 0.722) (Figure 4C) and peripheral (*F*(2,26) = 2.445; *p* = 0.106) (Figure 4D) areas were analyzed separately (Figure 4B).

### Acoustic startle response

**Figure 5:**
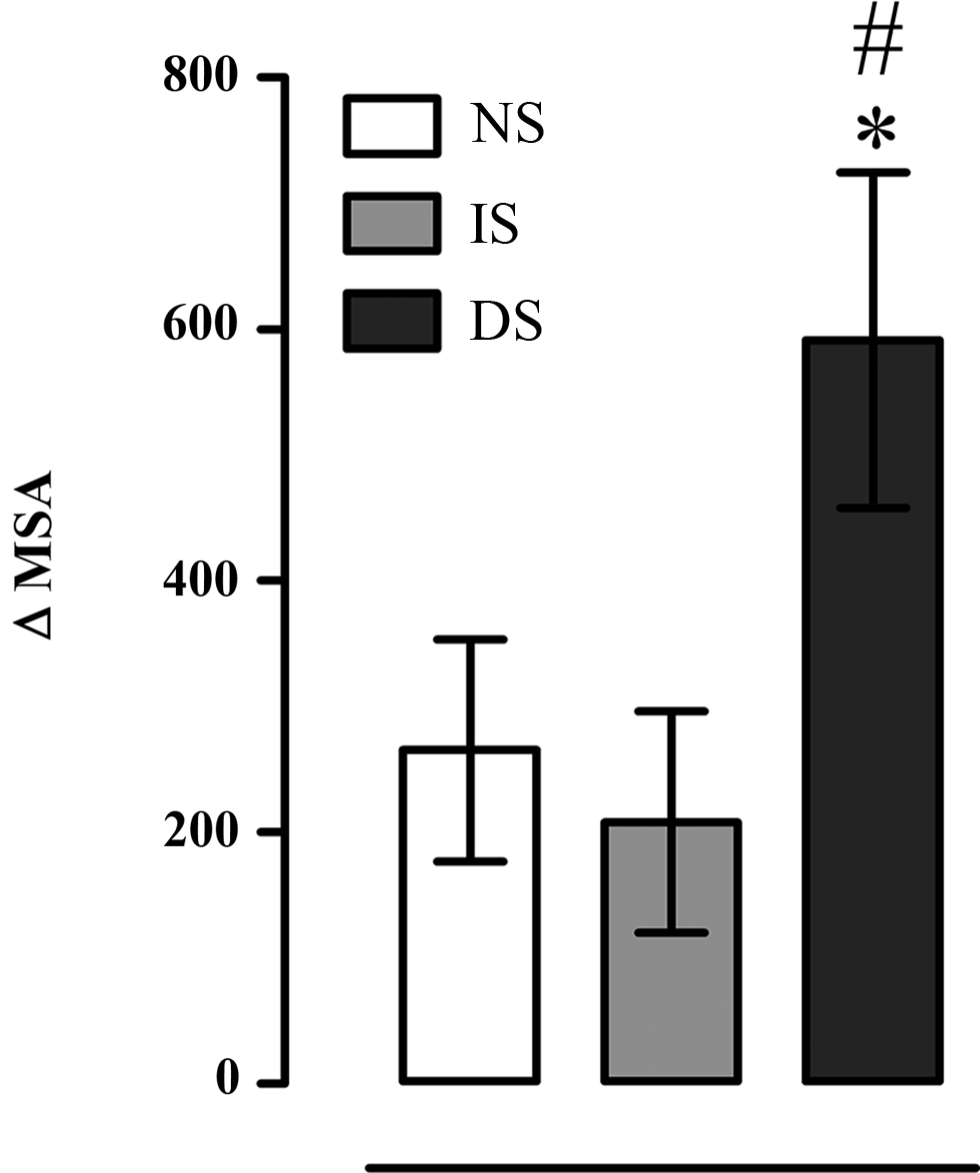
**Difference in mean startle amplitude between moments with presence and absence of pulse (Δ MSA); NS = no-shock group (*n* = 10); IS = immediate shock group (*n* = 10); DS = delayed shock group (*n* = 10).** *p < 0.05 compared to NS group; ^#^*p* < 0.05 compared to IS group.

There was a significant effect on Δ (MSA) (*F*(2, 27) = 3.853, *p* = 0.033). *Post-hoc* analysis revealed that with pulse presentation DS group displayed higher magnitude of the Δ MSA than NS (*p* = 0.037) and IS groups (*p* = 0.040) (Figure 5).

## Discussion

In animal models of traumatic stress based on contextual fear conditioning, the location where the aversive event occurs is assimilated by gathering multimodal stimuli and integrating all these environmental characteristics into a unique contextual representation, which is then associated with an aversive unconditioned stimulus, generally an electric foot shock (Sanders et al., 2003; Rudy et al., 2004). In the present study, environment exploration prior to the aversive stimulus was essential for the development of long-term emotional disturbance, characterized by inhibition of exploratory activity, fear sensitization and hyperarousal. We found that long-term emotional alterations due to the aversive event were present only in rats that had sufficient time to explore the context prior to the foot shock.

Classically, rats that receive immediate foot shock do not display the typical conditioned fear response upon re-exposure to the context, whereas those allowed to explore the chamber by delaying the foot shock presentation for, at least, 27 sec display retention of the contextual fear conditioning when re-exposed to the chamber (Fanselow, 1986; Landeira-Fernandez et al., 2006). Here, we found a weaker fear expression upon re-exposure to the conditioning chamber in the IS group, compared to the DS group. Analyzing minute by minute freezing upon re-exposure, we found a comparable initial fear reaction to the conditioned context for the groups, but the reaction declined throughout the session in the IS group only. Despite this clear, albeit transient reaction to the context, the IS group did not exhibit as much inhibition of exploratory activity, fear sensitization or hyperarousal as the DS group in the subsequent emotional assessments. The minute by minute results suggest a frail conditioned contextual fear reaction in the IS group, which is subjected to intra-session suppression, while fear is maintained in the DS group throughout the whole session.

It is plausible to assume that re-exposure might be affecting the direction of alterations observed in succeeding behavioral assessments. In other words, animals that present an intra-session fear suppression (IS group) show no behavioral sequelae, opposed to what happens to those animals showing no intra-session fear suppression (DS group). In fact, it was demonstrated that re-exposure to situational reminders can contribute to the behavioral alterations typically observed in PTSD animal models (Pynoos et al., 1996; Pawlyk et al., 2005; Diehl et al., 2012). However, in early pilot non-published experiments that lead to the present study, re-exposure was performed only after all behavioral assessments, and a similar pattern of emotional outcomes was found. On the other hand, to directly address this question, it would be necessary to elaborate an experimental design composed by re-exposed and non-exposed animals to compare the long-term outcomes. Nevertheless, it is beyond the scope of the present work to assess the influence of re-exposure on the subsequent alterations described. From the present data, we can only assume that DS group shows a more robust contextual fear response, resistant to re-exposure suppression, and display deleterious emotional consequences.

On the elevated plus maze, the DS group showed a reduced exploration of closed, but not open arms compared to the other groups. Classically, the measure for anxiety-like behavior is the number of entries in the open arms of the EPM (Pellow et al., 1985; Belzung and Griebel, 2001; Wall and Messier, 2001; Carobrez and Bertoglio, 2005), which was not affected in this study. Because time spent in each arm did not differ among groups, we believe that reduced number of closed arm entries observed in the DS group might have, most likely, reflected decreased ambulation. The evaluation of traveled squares confirmed this hypothesis, supporting the proposition that exploratory drive might be affected rather than an effect on anxiety behavior. Immobility on the EPM was markedly superior in DS compared to IS and NS rats. This result suggests that general exploratory behavior was impaired. Impaired exploration parallels behavioral blunting, which represents a general avoidance reaction, also regarded as a core feature in PTSD patients (Charney et al., 1993; Breslau et al., 2005; North et al., 2009).

Regarding the sensitized fear reaction, several studies have reported fear sensitization as an index of emotional sequelae in animal models of PTSD (Siegmund and Wotjak, 2007a,b; Golub et al., 2009; Siegmund et al., 2009; Pamplona et al., 2011). In the present study, sensitization was consistent across behavioral tests. Both in the unknown circular arena and the startle assessment, only the DS group showed exacerbation of freezing behavior and augmented startle amplitude in the presence of arousing acoustic stimuli. Taken together, these results illustrate the assembly of behavioral alterations exclusive to the DS group, suggesting that previous contextual exploration, and not merely shock presentation influenced behavioral sensitization in the long-term.

It is tempting to explore why context familiarization would affect so strongly the long-term outcomes of an aversive event. One first possibility could be the differential emotional states experienced during the event. During contextual exploration, animals play an active role in contextual learning, by being constantly attentive to all aspects of their environment, seeking for information that may become significant in case of emotionally relevant events (Mackintosh, 1965). It is possible that during this active exploration period, the animal detects that the environment is enclosed and there are no means of escaping. In 1976, Blanchard and co-workers touched this issue, hypothesizing that the difference in freezing expression observed immediately after shock delivery could arise from distinct sensations triggered by knowledge about inescapability of the conditioning chamber. According to their view, rats receiving immediate foot shock adopt an active search for means to escape, in contrast to delayed shock animals, which adopt a helpless posture, since they have previously learned that there is no way of escaping (Blanchard et al., 1976). Therefore, the hopelessness resulting from the inability to escape during the traumatic event may elicit the long-term emotional changes observed in DS rats. Conversely, trauma unaccompanied by learning about the impossibility of escaping the situation would cause only a moderate and adaptive state of alert, which allows the individual to avoid or escape future aversive situations, without, however, taking extreme states of behavioral inhibition, fear and hypervigilance.

Although assessing psychological states in animal models may seem a speculative matter, the physiological state experienced by the two groups might be a tangible candidate to underlie the differential long-term effects. The time spent in the conditioning chamber differs between DS and IS groups. Delaying shock delivery by 2 min also means prolonging exposure to a novel unknown environment. When foot shock is delivered, the time course of noradrenaline and corticosterone release might be different in these two groups. The neurophysiological effects of stress exposure, that ultimately affect memory, are well-known to involve a synergic corticosteroid and noradrenergic action in the amygdala, a core region for the associative memory formation (Quirarte et al., 1997; Roozendaal et al., 1999, 2002; Ledoux, 2000), which is believed to be timing dependent (Joels et al., 2011). Additionally, amygdala hyperactivity is supposed to underlie the long-term enhanced emotionality in PTSD (Bremner et al., 1999; Lanius et al., 2001, 2003; Shin et al., 2006; Williams et al., 2006). The divergent neuronal peritraumatic milieu between IS and DS groups might, hence, permanently affect the amygdala in different ways, which would prompt the differential behavioral outcomes here observed.

Alternatively, re-experiencing reactions might underlie the altered emotional behavior found in the present study. PTSD has been extensively claimed as a memory disorder. The maladaptive long-term reactions are generally triggered by event-related cues or internal states that prime re-experiencing of the traumatic event through intrusive flashbacks or nightmares (DSM-IV, 2000). One possible explanation for the phenomenon that we found would be that exposure to novel or potentially threatening situations would engender an aroused emotional state, which in turn, would prime the retrieval of the traumatic experience, through the well-consolidated contextual memory (in the DS group only); this would trigger exaggerated fear reactions, as it happens in PTSD patients. However, this is a somewhat theoretical assumption, since there is no testable method to assess subjective memory retrieval in non-human animals.

Although any of the possible explanations cannot be conclusive, the present study brings a novel animal approach to address memory processing in PTSD. Much attention has been directed to potential factors determining vulnerability and resilience to psychological disorders, especially those like PTSD, triggered by unpredictable and devastating events. The intriguing question that rises from the present data concerns the underlying mechanisms for the differential emotional effects of foot shock showed by DS and IS groups. It is appealing to explore distinctive psychological and neurobiological features between these groups in order to search for explanations that might help elucidate the differentiation between victims that develop and victims who do not develop PTSD, based on their memory trace for an event. Our animal paradigm represents a novel approach to investigate the relevance of contextual memory strength for the development of PTSD.

## Conflict of interest statement

The authors declare that the research was conducted in the absence of any commercial or financial relationships that could be construed as a potential conflict of interest.
